# Recent Advances in the Use of Focused Ultrasound as a Treatment for Epilepsy

**DOI:** 10.3389/fnins.2022.886584

**Published:** 2022-06-20

**Authors:** Emma Lescrauwaet, Kristl Vonck, Mathieu Sprengers, Robrecht Raedt, Debby Klooster, Evelien Carrette, Paul Boon

**Affiliations:** ^1^4Brain Lab, Department of Neurology, Ghent University Hospital, Ghent, Belgium; ^2^Department of Electrical Engineering, Eindhoven University of Technology, Eindhoven, Netherlands

**Keywords:** neuromodulation, non-invasive, refractory epilepsy, low intensity focused ultrasound, high intensity focused ultrasound

## Abstract

Epilepsy affects about 1% of the population. Approximately one third of patients with epilepsy are drug-resistant (DRE). Resective surgery is an effective treatment for DRE, yet invasive, and not all DRE patients are suitable resective surgery candidates. Focused ultrasound, a novel non-invasive neurointerventional method is currently under investigation as a treatment alternative for DRE. By emitting one or more ultrasound waves, FUS can target structures in the brain at millimeter resolution. High intensity focused ultrasound (HIFU) leads to ablation of tissue and could therefore serve as a non-invasive alternative for resective surgery. It is currently under investigation in clinical trials following the approval of HIFU for essential tremor and Parkinson’s disease. Low intensity focused ultrasound (LIFU) can modulate neuronal activity and could be used to lower cortical neuronal hyper-excitability in epilepsy patients in a non-invasive manner. The seizure-suppressive effect of LIFU has been studied in several preclinical trials, showing promising results. Further investigations are required to demonstrate translation of preclinical results to human subjects.

## Introduction

Epilepsy is a highly prevalent neurological condition, affecting about 1% of the population worldwide (de Boer et al., 2008; Boon et al., 2018). In epilepsy patients, the balance between excitation and inhibition in the brain is disrupted. Groups of neurons become hyperexcitable, leading to a state of recurrent, spontaneous seizures (Scharfman, 2007). Antiepileptic drugs (AEDs) are the first line of treatment to reduce the excitability of the brain and thereby restore the balance and lower the seizure frequency. Despite extensive research in novel AEDs over the past decades, drug failure still occurs in 25–30% of the epilepsy population (López González et al., 2015).

For this group of drug-resistant epilepsy (DRE) patients, resective epilepsy surgery is the most effective treatment option following a thorough presurgical evaluation (Englot and Chang, 2014). Despite the invasiveness of the procedure, it is regarded as a safe and effective technique (Wiebe, 2004). Unfortunately, up to 60% of DRE patients are considered unsuitable for surgery due to the existence of the epileptogenic focus in functional tissue or due to the inability of defining a unique epileptogenic zone. Over the past two decades, neurostimulation techniques as a treatment for DRE have gained more interest. Vagus nerve stimulation (VNS), deep brain stimulation (DBS), and responsive neurostimulation (RNS) are invasive with accompanying risks (Elliott et al., 2011; Hartshorn and Jobst, 2018; Toffa et al., 2020; Foutz and Wong, 2021). Non-invasive neuromodulation techniques allow to treat patients without any incision and have a lower risk for surgery related side effects. Non-invasive cranial nerve stimulation [trigeminal nerve stimulation (TNS), transcutaneous vagus nerve stimulation (tVNS)], repetitive transcranial magnetic stimulation (rTMS) or transcranial direct current stimulation (tDCS) are currently investigated as a potential treatment for epilepsy (Pack, 2013; Bauer et al., 2016; Cooper et al., 2017; San-Juan et al., 2017; Gil-López et al., 2020). However, these techniques have a low spatial specificity and limited depth of penetration (Tergau et al., 1999; Berényi et al., 2012; DeGiorgio et al., 2013). Table 1 gives an overview of the currently available treatments and treatment options under investigation for DRE patients.

**TABLE 1:** An overview of the currently available treatments and treatment options under investigation for DRE patients.

	Epilepsy surgery	VNS	DBS	RNS	eTNS	rTMS	tDCS	tVNS	FUS
Responder rate°,*	±70%	45–65%	±70%	±65%	30–50%	±30%	±50%	25–30%	NA
FDA approved for epilepsy	NA	Yes	Yes	Yes	No	No	No	No	No
Invasiveness	High	Moderate	High	High	No	No	No	No	No
Spatial targeting resolution	High	NA	High	NA	NA	Low	Low	Low	High
Targetable brain regions*	Deep and superficial cortex (determined by target location)	NA	Deep and superficial cortex	NA	NA	Superficial cortex ∼1–3.5 cm	Undefined	NA	Deep cortex ∼10–15 cm
References	Englot and Chang, 2014	Elliott et al., 2011; Toffa et al., 2020	Salanova, 2018; Foutz and Wong, 2021	Hartshorn and Jobst, 2018	DeGiorgio et al., 2013; Pack, 2013; Gil-López et al., 2020	Bystritsky et al., 2011; Deng et al., 2013, 2014; Cooper et al., 2017	San-Juan et al., 2017	Bauer et al., 2016	Bystritsky et al., 2011

Responder rate = the percentage of patients with at least 50% seizure frequency reduction, °for epilepsy surgery percentage of patients who become seizure-free. VNS, vagus nerve stimulation; DBS, deep brain stimulation; RNS, responsive neurostimulation; eTNS, external trigeminal nerve stimulation; rTMS, repetitive transcranial magnetic stimulation; tVNS, transcutaneous vagus nerve stimulation; FUS, focused ultrasound; NA, not applicable. *Numbers reported in systematic reviews or derived from recent trials (cfr. References in last row).

Focused ultrasound (FUS) is a novel and promising treatment method for neuropsychiatric disorders. This method uses one or more ultrasound beams at either a low or high intensity to respectively modulate brain activity or ablate neuronal tissue. These beams are high pressure waves that are emitted by a pulse generator and amplified by a transducer. Directing the beam(s) toward a focal point in the brain leads to acoustic energy at the target site. FUS is often used in combination with magnetic resonance imaging (MRI) guidance to define the target tissue at millimeter resolution and to evaluate lesioning effects during the FUS procedure (Hectors et al., 2016). A sonication protocol contains five parameters: the fundamental frequency (FF), pulse repetition frequency (PRF), duty cycle (DC), sonication duration, and intensity (Fomenko et al., 2018). Adjusting these parameters can influence the nature, magnitude and spatial specificity of the effect (King et al., 2013; Younan et al., 2013).

Focused ultrasound has some major potential benefits compared to other non-invasive techniques. It allows targeting of deeper brain structures without damaging surrounding non-target tissue. When FUS is combined with MRI guidance, the tissue can be focally targeted with high spatial precision (Hynynen and Jolesz, 1998; McDannold et al., 1998; Bystritsky et al., 2011). The non-ionizing nature of FUS allows to repeat the therapy when required (Elhelf et al., 2018). Concerning HIFU, sub-ablative treatment parameters can be used to specify the target prior to ablation. To date, HIFU is FDA approved for essential tremor and Parkinson’s disease, as well as several non-neurological disorders. Due to its high potential, FUS is currently extensively researched as a treatment for other neurological and non-neurological disorders, including epilepsy.

### Focused Ultrasound as a Treatment for Epilepsy

Focused ultrasound could potentially serve as a non-invasive and safe method to lesion the epileptic zone or target epilepsy networks or foci in a neuromodulatory way in DRE patients.

#### Low vs. High Intensity Focused Ultrasound

**FIGURE 1:**
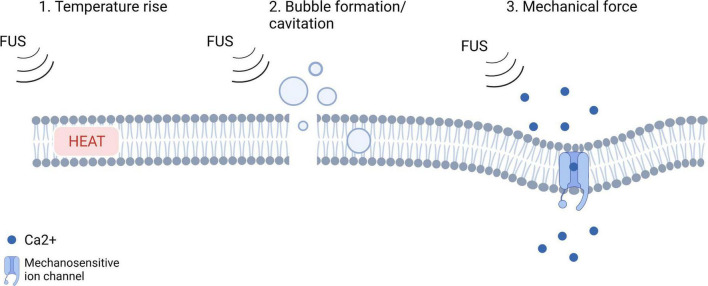
An illustration of the hypotheses regarding the mechanism of action of LIFU (adjusted from Yoo et al., 2022; Created with BioRender.com).

One of the most dominant parameters of the sonication protocol is the intensity. With low intensity focused ultrasound (LIFU), the emitted beams induce reversible mechanical effects on a cellular level (Baek et al., 2017). LIFU has bimodal capabilities, as it can both excite or inhibit neural activity within a specific brain region (Baek et al., 2017). To date, it is unclear what mechanisms underly these phenomena. Figure 1 illustrates the hypotheses regarding the mechanism of action of LIFU. Heating caused by the absorption of acoustic energy could disrupt synaptic signaling in the targeted tissue. Several preclinical studies monitored the temperature at the sonication target using a fiber optic thermometer and reported that heat increase caused by LIFU is low (<0.1°C) (Tufail et al., 2010; Yoo et al., 2011, 2022; Baek et al., 2017). A study by Yoo et al. (2022) investigated the thermal effects of LIFU by using the fluorescent protein mCherry42 as a temperature indicator. The mCherry fluorescence remained unchanged while neurons responded to ultrasound, indicating that there was no significant temperature rise at the sonication target (Yoo et al., 2022). Heating *per se* is therefore not believed to underly the mechanism of action of LIFU but further research is needed to confirm this. Changes in membrane capacitance have been investigated to estimate the occurrence of cavitation of the cell bilayer as underlying phenomenon but were found to be minimal or absent following LIFU (Rohr and Rooney, 1978; Krasovitski et al., 2011; Prieto et al., 2013; Plaksin et al., 2014). The majority of currently published research supports the hypothesis that LIFU mechanically deforms mechanosensitive ion channels embedded within cellular membranes (Tyler, 2012; Baek et al., 2017). This could lead to a higher probability of channel opening and ion influx, resulting in depolarization of the cell and the activation of voltage-gated ion channels, which in turn could generate action potentials.

Kubanek et al. (2016) investigated the expression of potassium and sodium mechanosensitive ion channels in the Xenopus oocyte system (Kubanek et al., 2016). They showed that LIFU modulated the currents flowing through the ion channels on average by up to 23%, depending on channel and stimulus intensity. After adding a channel blocker to the solution, these effects were no longer observed. In another study, they investigated the mechanistic hypothesis by administering LIFU to both thermosensory and mechanosensory mutants of Caenorhabditis elegans nematodes. They found that thermosensory mutants responded to ultrasound similarly to wild-type animals, but that mechanosensory mutants were insensitive to ultrasound stimulation. Additionally, stimulus parameters that accentuate mechanical effects were more effective than those producing more heat (Kubanek et al., 2018). Further evidence for the mechanistic explanation for the effects of LIFU was provided by a study by Yoo et al. (2022) demonstrating that an overexpression of mechano-sensitive ion channels resulted in strong effects of LIFU, whereas inhibiting these channels led to reduced responses to ultrasound (Yoo et al., 2022).

In high intensity focused ultrasound (HIFU) the emitted beams have a far higher intensity, with a spatial peak pulse average >100 W/cm^2^. With HIFU, neuronal tissue will be ablated rather than modulated. The absorption of the acoustic energy results in heat, leading to a rapid temperature increase of up to 60°C or higher. This increase leads to coagulation necrosis in a short moment of time. The created lesion typically has a cigar shape and is as big as a rice grain (Elhelf et al., 2018). To precisely lesion the target tissue and to evaluate the ablation effect of HIFU during treatment, HIFU is usually MRI-guided (Hectors et al., 2016). The mechanism of action is based on the interaction of the acoustic beams with so called micro gas bubbles that are present in the targeted tissue. A steady oscillation of the bubbles is caused, leading to a local shear force. Due to this, the temperature at the focus mildly elevates leading to a pressure increase. As soon as the bubbles reach a certain pressure threshold, they collapse. This results in a higher temperature rise and strong pressure wave (Shehata, 2012).

Both low and high intensity focused ultrasound are potentially effective in the treatment of refractory epilepsy, as it could either modulate or ablate the epileptic focus. In this paper, we aim to provide an overview of the published studies so far, investigating the effect of low and high intensity FUS on epilepsy.

## Methodology

We searched online databases (Pubmed, ScienceDirect, clingov) and preprint servers^1, 2^ for the combination of focused ultrasound [high intensity focused ultrasound terminology (HIFU), low intensity focused ultrasound (LIFU), MRI-guided focused ultrasound (MRgFUS)] and epilepsy terminology [epilepsy, drug-resistant epilepsy (DRE), refractory epilepsy] up to 20 February 2022. The Focused Ultrasound Foundation news page and social media channels were followed up for postings with the same scope. All relevant papers testing the safety and/or efficacy of LIFU or HIFU in animal models for epilepsy or human subjects were included in this review.

## High Intensity Focused Ultrasound

The effects of HIFU have been tested in one preclinical study dating from 1964 using epileptic cats. Epilepsy was induced by injecting Alumina cream subcortically. The effectiveness of HIFU treatment, resective surgery and medical treatment on the seizure frequency and EEG patterns was compared. HIFU was targeted 2 mm below the injection site of the alumina cream, which was either the middle suprasylvian gyrus or the anterior sigmoid gyrus. The dose of sonication was calculated to create a lesion 15 mm long and 5 mm in diameter. There were no further specifications about the device or stimulation parameters reported. It was shown that both HIFU and resective surgery led to abolition of EEG spike activity. Eight out of the nine surviving cats became seizure free after surgery, whereas 9 out of 11 became seizure free after HIFU. Six cats died after resective surgery due to post-operative complications, whereas only one cat died after HIFU. Medical treatment was not found to be effective, as none of the cats became seizure free after this treatment (Manlapaz et al., 1964).

To date only two case reports have been published in which MRI-guided HIFU (MRgFUS) was tested as a treatment in epilepsy patients (Abe et al., 2020; Yamaguchi et al., 2020). Yamaguchi and colleagues reported the effects of MRgFUS in a 26-year-old man with gelastic epilepsy who had been diagnosed with hypothalamic hamartoma (HH). MRgFUS ablation was targeted to the boundary area of the HH to disconnect the hamartoma cells from the base of the hypothalamus. Six therapeutic sonications at 50–53°C were administered, no further specifications about the used sonication parameters were reported. An MRI scan 1 day after the treatment demonstrated an oval-shaped lesion at the boundary area. At 1-year follow-up, seizure frequency had dropped significantly from 2 seizure per month pre-treatment to seizure freedom post-treatment in addition to a decrease in AED dosage (400 mg of Carbamazepine per day preoperatively to 50 mg per day at 1-year) (Yamaguchi et al., 2020).

A trial conducted by Abe et al. (2020) administered MRI-guided HIFU to a female patient with left temporal lobe epilepsy. Twelve sonication sessions, with a duration of 10–20 s, were performed targeting the hippocampus. The temperature at the target site was monitored in real-time using MRI. The sonication led to a temperature increase up to 48°C of the target tissue, which was lower than the desired ablation temperature (>54°C). MRI 1 month after HIFU application did not show any lesion. However, the seizure frequency decreased from 3 to 4 seizures a month to almost seizure freedom for 12 months after treatment, despite an unchanged AED regimen (Levetiracetam – 1000 mg/day). This finding supported the idea that HIFU may induce neuronal changes that could either be less or different than those induced by typical ablation, but sufficient to possibly induce physiological changes to affect regional seizure threshold. Small lesions caused by the sonication but undetectable with MRI is an alternative explanation to be considered. The inability to reach the target temperature in this study was probably due to the small number of transducer elements and incident angles applied. A study using cadaveric skulls showed that a longer sonication duration (e.g., 30 s) is required to reach a sufficient temperature rise for permanent lesioning at the target site. These longer sonication durations may lead to more heating at the skull base (up to 24.7°C) (Montheith et al., 2016).

Currently, there are two ongoing clinical trials investigating the feasibility and safety of HIFU in epilepsy patients. One trial focuses on using HIFU to ablate the anterior nucleus of the thalamus to prevent secondary generalization in focal onset epilepsy. Another trial investigates the effects of ablating the anterior nucleus in epilepsy patients with comorbid moderate-severe anxiety (National Library of Medicine, 2018, 2022b). In Table 2, a schematic overview of clinical HIFU protocols is provided.

**TABLE 2:** Schematic overview of case reports and ongoing clinical trials with HIFU in epilepsy patients.

Author/Study nr.	Epilepsy type	Sample size	HIFU parameters	Target	Main results/Objectives
Abe et al., 2020	Mesial TLE	*N* = 1	Repetitive, low power, 10–20 s, 42–44°C	Hippocampus	Desired ablation temperature not reached, no lesion observed ↓ Seizure frequency
Yamaguchi et al., 2020	Gelastic epilepsy caused by hypothalamic hamartoma	*N* = 1	Six sonications at 50–53°C	5 target sites at boundary area of the HH	Lesion observed at target Seizure freedom after 1-year follow-up
NCT03417297 (recruiting)	Partial seizures with secondary generalization	*N* = 10	NR	Anterior thalamic nucleus	Safety and feasibility of HIFU in epilepsy patients
NCT05032105 (not yet recruiting)	Epilepsy patients with comorbid anxiety	*N* = 10	NR	Anterior thalamic nucleus	Safety and feasibility of HIFU effect of HIFU on anxiety

*HIFU, high intensity focused ultrasound; TLE, temporal lobe epilepsy; NR, not reported; HH, hypothalamic hamartoma.*

## Low Intensity Focused Ultrasound

In contrast to HIFU, LIFU has been more extensively researched as a treatment for epilepsy in the past decade. LIFU could serve as a non-invasive technique to decrease the cortical excitability and thereby lower seizure frequency, without damaging neuronal tissue. Several animal studies have been performed to investigate the efficacy and safety of LIFU, with promising results. Table 3 provides a schematic overview of preclinical trials investigating behavioral and neurophysiological effects of LIFU in experimental epilepsy models.

**TABLE 3:** Schematic overview of preclinical trials investigating behavioral and neurophysiological effects of LIFU in experimental epilepsy models.

Author	Year	Experimental model	Sample size	LIFU parameters	Target	Main results
Zhang et al.	2021a	Rats Acute KA	*N* = 21	FF: 0.5 MHz PRF: 1.5 kHz DC: NR Duration: NR Energy: max. 101.1 mW/cm^2^	Hippocampus	↓ EEG average amplitude ↓ Network connection strength
Zhang et al.	2021b	Rats Acute Pilocarpine	*N* = 30	FF: 0.65 MHz PRF: 1 Hz DC: 2% Duration: 90 s per sonication Energy: NR	Hippocampus	↓ Seizure frequency after administering a neurotoxin by opening the BBB Elimination of convulsive seizures in two animals
Zhang et al.	2021c	Rats Acute KA	*N* = 27	FF: 0.25–0.65 MHz PRF: 1.5 kHz DC: NR Duration: 40 s Energy: NR	Hippocampus	↓ EEG power spectral density and connection strength of the brain network after administering two modes of LIFU No significant difference between the two modes.
Zhang et al.	2020	Mice Acute Pilocarpine	*N* = 11	FF: 1.5 MHz PRF: 1 Hz DC: 2% Duration: 120 s per sonication Energy: NR	Hippocampus	↓ Behavioral seizure of 21.2% after administering a neurotoxin by opening the BBB
Lin et al.	2020	Monkeys Acute Penicilin	*N* = 5	FF: 0.75 MHz PRF: 100 Hz DC: NR Duration: 1 × 30 min Energy: Ispta: 233 mW/cm^2^ Isppa 2.02 W/cm^2^	Temporal lobe, not further specified	↓ Seizure frequency
Zou et al.	2020	Rhesus monkeys Acute Penicilin	*N* = 2	FF: 0.8 MHz PRF: 500 Hz DC: 36% Duration: 1 × 15 min Energy: NR	Right hand movement area	↓ Seizure frequency
Chen et al.	2019	Rats Acute Pentylenetetrazol	*N* = 76	FF: 0.5 MHz PRF: 100 Hz DC: 8%, 30% Duration: 1 × 10 min Energy: 0–2.812 W/cm^2^	Hippocampus and thalamus regions	↓ Epileptic activity Expression level changes of c-FOS and GAD65
Li et al.	2019a	Mice Acute KA	*N* = 37	FF: 0.5 MHz PRF: 500 Hz DC: 50% Duration: 30 s, per seizure Energy: NR	Hippocampus (CA3)	↓ LFP intensity in the low frequency (<10 Hz) bands ↑ inter-seizure interval
Li et al.	2019b	Mice Acute KA	*N* = 14	FF: 0.5 MHz PRF: 500 Hz DC: 50% Duration: 30 s, per seizure Energy: NR	Hippocampus (CA3)	↓ Seizure frequency ↑ Complexity, approximate entropy of the delta/theta frequency bands, and Lyapunov exponent of the LFP
Hakimova et al.	2015	Mice Acute KA	*N* = 34	FF: 0.2 MHz PRF: 500 Hz DC: NR Duration: 30 s, per seizure Energy: NR	Hippocampus	↓ Acute seizures Improvement in behavioral task
Min et al.	2011	Rats Acute Pentylenetetrazol	*N* = 27	FF: 0.69 MHz PRF: 100 Hz DC: NR Duration: 2 × 3 min. Energy: 130 mW/cm^2^	Thalamus	↓ EEG burst activity

*KA, kainic acid; NR, not reported; FF, fundamental frequency; PRF, pulse repetition frequency; DC, duty cycle; LFP, local field potential; BBB, blood–brain barrier; LIFU, low intensity focused ultrasound.*

A first preclinical study investigating the neurophysiological and biological effects of LIFU in epileptic rats was performed by Min et al. (2011). Epileptic rats were treated with or without FUS and a healthy control group also underwent FUS. LIFU was administered two times for 3 min (spatial peak temporal average intensity of 130 mW/cm^2^) targeting the thalamus. Before and after two FUS interventions, subdermal EEG was recorded for 10 min to evaluate neurophysiological effects and behavioral monitoring was performed. Results showed that epileptic bursts were significantly reduced after the first period of sonication, and even further decreased after the second sonication. This effect was not observed in the control epileptic group who did not receive FUS treatment. The Racine score used to evaluate seizure severity in experimental models of epilepsy on the day after the experiment was remarkably lower in the group treated with FUS compared to the unsonicated epileptic group. A histological analysis was performed on the non-epileptic treated rats and confirmed that there was no tissue damage induced by the sonication, indicating that FUS could be safely delivered to the target region (Min et al., 2011). The seizure-suppressive effect of LIFU was later confirmed in other rodent studies (Hakimova et al., 2015; Chen et al., 2019; Li et al., 2019a; Zhang et al., 2021a, b,c).

The neurophysiological effects of LIFU were further explored over the years. Chen et al. (2019) found that the expression level of c-FOS, an indirect marker of neuronal activity, and GAD65, an indirect marker of GABAergic neurons, were significantly altered following FUS administration. As one of the potential explanations for a significant decrease of c-fos is an attenuation of neuronal activity, the authors concluded that sonication with these specific parameters (frequency: 0.5 MHz, duration: 10 min, energy: 0–2.812 W/cm^2^) has the potential to affect excitatory cells. In this study a significant increase of GAD65 was also found in the cortex of sonicated rats further supporting potential inhibitory effects of LIFU in this study. Li et al. (2019b) investigated whether and how ultrasound is able to modulate the non-linear dynamic characteristics of EEG signals in temporal lobe epilepsy by recording local field potentials before, during and after stimulation of the hippocampus in epileptic mice. Complexity, approximate entropy of different frequency bands, and Lyapunov exponent of the local field potential were calculated as outcome parameters as it was previously reported that these can be used as biomarkers of epileptic activity (Varatharajah et al., 2018). These parameters were described to have low values during epileptic activity (Osowski et al., 2007; Weng et al., 2015; Li et al., 2018; Raghu et al., 2018). Results of this study showed that LIFU inhibited TLE seizures in the experimental group. The complexity, approximate entropy of the delta (0.5–4 Hz) and theta (4–8 Hz) frequency bands, and Lyapunov exponent of the LFP were significantly increased after LIFU (Li et al., 2019b). In a functional connectivity study, the effect of LIFU on brain network was investigated by comparing the brain network before and after administering LIFU in epileptic rats. Apart from a decrease in average EEG amplitude after LIFU, it was seen that LIFU significantly decreased the brain network connection strength across multiple brain regions. This effect was especially prominent in the theta band. Therefore, this was proposed as an alternative hypothesis for the underlying working mechanisms of LIFU. LIFU would be able to control neural circuits by affecting functional connections in the brain. Based on this, it is believed that LIFU could serve as a method to reduce the strength of the epileptic network and thereby lower the seizure frequency (Zhang et al., 2021a,c).

Besides neuromodulation, LIFU can also be used to temporarily open the blood brain barrier (BBB). In this way, drugs can be specifically targeted toward certain brain regions. Zhang et al. (2020) studied the seizure suppressive effect of administering a neurotoxin to the hippocampus using LIFU. In epileptic mice, the BBB at the hippocampus site was opened using MRI-guided LIFU and the neurotoxin Quinolinic acid was administered. Neuronal loss was detected in 8 out of 11 mice. The seizure frequency in these mice was reduced by 21.2% (Zhang et al., 2020). Later, the effectiveness of this method to lower the seizure frequency was confirmed in a controlled trial using rat models (Zhang et al., 2021b). In addition to neurophysiological effects, it has been shown that LIFU also affected behavior in epileptic mice, as LIFU significantly improved sociability, reflected by an increase in the time spent with an unfamiliar mouse, and depressive behavior, measured by the forced swim task, compared to non-sonicated epileptic animals (Hakimova et al., 2015).

In all aforementioned studies the effect of LIFU was tested in rodent models. However, the ultimate goal is to eventually apply this technique in human subjects. Therefore, the translational potential of the preclinical findings needs to be confirmed. As a first step in doing so, Lin et al. (2020) investigated the effects of LIFU in non-human primates. Thirty minutes of LIFU stimulation was administered to epileptic monkeys, while the neurophysiological effects were recorded with depth electrodes during 8 h following sonication. Different behavioral seizure parameters (total seizure count, seizure frequency per hour, seizure duration and seizure interval time) were measured during 16 h after sonication. It was shown that LIFU significantly decreased ictal spiking activity and significantly reduced all aforementioned behavioral seizure parameters, except for the seizure interval time which was increased in these epileptic monkeys (Lin et al., 2020). Similar results were later reported by Zou et al. (2020) by targeting LIFU to the right hand motor area for 15 min in an acute monkey model (Zou et al., 2020). Both studies confirm that LIFU seems to be effective in higher-order animals and thereby paved the way for translation of this neuromodulation technique to humans.

Only one clinical trial investigating the effects of LIFU in epileptic patients has been published so far (Lee et al., 2021). This phase 1 open label uncontrolled trial aimed to investigate both the efficacy and safety of LIFU in DRE patients. Six patients with a seizure frequency ranging between two seizures per month to three events per day were included in this study. All patients underwent stereo-encephalography (SEEG) to localize their seizure onset zone (SOZ). The hypothesized SOZs, based on clinical data, imaging data and neuropsychological investigation were different in each patient and guided the sonication targets. LIFU was administered for 10 min at an intensity of 2.8 W/cm^2^. The transducer was directed at the SOZ under the real-time guidance of the neuronavigation system. SEEG recordings were performed before, during and after LIFU. To assess the potential seizure suppressive effects of LIFU, the clinical seizure frequency and frequency of interictal epileptiform discharges (IEDs) within 24 h before treatment were compared to those within 72 h after treatment.

Due to the low baseline seizure frequency, no seizures were detected before and after LIFU in three out of six patients. In the remaining three patients, seizures were recorded before treatment and within 72 h posttreatment. In two of these patients, the seizure frequency was decreased, whereas one patient showed a seizure frequency increase. Concerning the recorded IEDs, four patients showed a decrease in IED frequency and two patients showed an increase. Based on the SEEG recordings before, during and after treatment, an effect of LIFU was solely detected in the electrode contacts at the target site. In two patients, a significant decrease in spectral power was detected in all frequency bands after LIFU. However, no correlation between these short-duration effects and the seizure frequency could be established. In one patient, a significant decrease in EEG band power could only be detected in the theta band, no change was seen in other frequency bands. An increase of EEG band power was detected in one patient, but only in the beta band. In the remaining two patients, no change in EEG band power was detected after LIFU.

Regarding safety, this study concluded that LIFU can be safely delivered to DRE patients. No radiological changes were observed in the posttreatment MRI scans. The cortical lamination was normal and no focal edema was observed in the cerebral white matter. Only two transient adverse events were reported. In one patient, uncomfortable scalp heating occurred during the treatment. After 1 h, a second treatment could be conducted without any complications. In another patient, impairment in naming and memory was experienced after FUS, but completely resolved after 3 weeks. No evidence of continuous slowing or non-convulsive seizures was found, but the exact etiology of this symptom remained unclear. Overall, this study suggests that LIFU can affect neural activity, without damaging tissue or structural lesioning. However, as this was a phase 1 study, no sham control was included and the sample size was limited (Lee et al., 2021). Currently, several ongoing trials are investigating the tolerability and effectiveness of LIFU in epilepsy patients (National Library of Medicine, 2014, 2019, 2022a). Table 4 provides an overview of published and ongoing clinical trials testing the effect of LIFU in epilepsy patients.

**TABLE 4:** Overview of published and ongoing clinical trials testing the effect of LIFU in epilepsy patients.

Author/Study nr.	Epilepsy type	Sample size	LIFU parameters	Target	Main results/Objectives
Lee et al., 2021	NR	*N* = 6	FF: NR PRF: 100 Hz DC: 30% Duration: 10 min Energy: <2.8 W/cm^2^	SOZ: Left fusiform gyrus, left premotor gyrus, right frontal operculum, left body of hippocampus, right superior border of insula, left anterior cingulate	↓ Spectral power in 1/3 of the patients ↓ Seizure frequency in two patients LIFU = safe in DRE patients
NCT03868293 (recruiting)	TLE	*N* = 10	NR	Epileptogenic focus (temporal region)	Adverse events assessment Efficacy of LIFU on seizure frequency Effect of LIFU on EEG
NCT03657056 (not yet recruiting)	TLE	*N* = 3	FF: NR PRF: 250 Hz DC: NR Duration: 2 min energy: 720 mW/cm^2^–5760 mW/cm^2^	Epileptogenic focus (temporal region)	Safety and feasibility of LIFU in DRE
NCT02151175 (enrolling by invitation)	TLE	*N* = 12	NR	Epileptogenic focus (temporal region)	Safety and efficacy of LIFU to stimulate or suppress brain activity in DRE

*NR, not reported; FF, fundamental frequency; PRF, pulse repetition frequency; DC, duty cycle; SOZ, seizure onset zone; LIFU, low intensity focused ultrasound; DRE, drug resistant epilepsy; TLE, temporal lobe epilepsy.*

The safety of LIFU has been evaluated in other studies, including both healthy subjects as well as epilepsy patients. In a study by Legon et al. (2020), the safety of LIFU was assessed by administering a follow-up participant report of symptoms questionnaire to 64 participants who underwent a LIFU neuromodulation experiment. No serious adverse events were reported. Only 11% of the participants reported mild to moderate symptoms that were perceived as ‘possibly’ or ‘probably’ related to participation in LIFU experiments. The most prevalent symptoms included neck pain, problems with attention, muscle twitches and anxiety. These initial symptoms disappeared upon follow-up (Legon et al., 2020). Studies investigating the effects of LIFU using TMS as an outcome measure in healthy subjects do not report any discomfort associated with the procedure, nor any mental or physical abnormalities assessed by follow-up neurological examinations and anatomical MRIs (Pasquinelli et al., 2019). Stern et al. (2021) assessed the safety of LIFU in eight TLE patients. Histological analysis did not reveal any damage after LIFU compared to before, except for one patient whose results were inconclusive. The results on neurophysiological testing were rather exploratory and inconclusive, due to the small sample size and the lack of a control group (Stern et al., 2021). It seems that when FDA safety guidelines [spatial peak temporal average intensity (Ispta) < 720 mW/cm^2^; spatial peak pulse average intensity < 190 W/cm^2^; mechanical index (MI) < 1.9; TI (thermal index) < 6] are properly followed, LIFU can be considered as a safe neuromodulation technique.

## Discussion

The goal of this review is to give an overview of the published preclinical and clinical trials investigating the potential of both low and high intensity FUS in the treatment of epilepsy. Concerning HIFU, only limited evidence is available. More preclinical and clinical research is needed to draw proper conclusions on its safety and effectiveness. The inability to attain desired ablation temperatures at deep targets is still a limitation to overcome (Abe et al., 2020). Overheating of the skull is a potential adverse event that needs to be avoided. Work in phantoms is required to optimize stimulation parameters and find a trade-off between sonication duration and skull heating before HIFU can be more extensively investigated in human subjects. So far, most studies have focused on targeting deep structures since the currently available HIFU technology is not suitable for targeting superficial cortex, a strategy that should be further developed with appropriate technical advancement.

Low intensity focused ultrasound has been more extensively studied the last few years. Although several preclinical studies support a mechanistic explanation for the effect of LIFU, more studies evaluating its mechanism of action on different levels are still needed to confirm this statement and rationally direct sonication therapy parameters. When investigating the effect of LIFU in epilepsy, various trials showed that LIFU can lead to a decrease in seizure frequency in epilepsy induced rodent models, indicating the potential of LIFU. However, given the paucity of trials, there is still limited evidence. Further investigations evaluating both the efficacy and safety are required to provide conclusive data. Despite the limited preclinical data, ongoing clinical trials are verifying whether the seizure suppressive effects of LIFU detected in animal models can be translated to human subjects. Lee et al. (2021) recently published a first pilot study testing LIFU in epilepsy patients. Although results were promising, the sample size was small and no control group was included. There is an unmet need for controlled clinical trials, with larger study groups and long-term follow up. In addition to clinical trials in patients, it would be interesting to further investigate the effects of LIFU in healthy volunteers. Up to date, there is no consensus on stimulation parameters and only little is known on how the adjustment of these parameters can influence the effects of FUS. To gain a better understanding of the mechanism of action of LIFU, LIFU has been tested using TMS-EMG in healthy subjects, showing that it suppresses TMS-elicited motor corticospinal activity and increases short-interval intracortical inhibition both during and after sonication (Fomenko et al., 2020; Xia et al., 2021). In addition to TMS-EMG, TMS-EEG could be used as to assess the effects of LIFU on cortical excitability in future studies, revealing the neurophysiological effects of LIFU in a more direct way. These studies may provide more insight in the potential of LIFU in the treatment of epilepsy. Apart from the neuromodulatory effects of LIFU on neuronal tissue, the capability of safely and reversibly opening the blood-brain barrier adds an additional therapeutic avenue by allowing targeted delivery of neurotherapeutics in neurological disorders, including epilepsy.

Overall, we can conclude that currently published studies report that focused ultrasound is a promising technique that may become an added value in the total therapeutic armamentarium for DRE patients who still suffer from an unsolved treatment gap. More preclinical research and clinical trials are necessary to unravel the exact mechanism of action and evaluate the efficacy and safety of FUS. In comparison to other available treatment techniques, FUS is non-invasive and allows to target deep structures at high spatial specificity (Table 1). However, at this time, it is too early to predict what techniques will be most suitable for individual DRE patients and appropriate protocols will have to be developed in analogy to the presurgical evaluation protocol and proposed pre-stimulation protocol for DRE (Carrette et al., 2017). The provided update on this novel non-invasive neurointerventional technique based on the currently available literature may serve as an opportunity to update neurologists, neurosurgeons as well as neuroscientists to increase awareness on the ongoing research with FUS, especially in the field of epilepsy.

## Author Contributions

EL wrote the initial draft of the manuscript. KV, MS, DK, RR, EC, and PB critically reviewed and edited the manuscript. All authors contributed to the article and approved the submitted version.

## Conflict of Interest

The authors declare that the research was conducted in the absence of any commercial or financial relationships that could be construed as a potential conflict of interest.

## Publisher’s Note

All claims expressed in this article are solely those of the authors and do not necessarily represent those of their affiliated organizations, or those of the publisher, the editors and the reviewers. Any product that may be evaluated in this article, or claim that may be made by its manufacturer, is not guaranteed or endorsed by the publisher.

## Footnotes

1
https://www.medrxiv.org/

2
https://www.biorxiv.org/
